# Navigating the Healthy Context Paradox: Identifying Classroom Characteristics that Improve the Psychological Adjustment of Bullying Victims

**DOI:** 10.1007/s10964-020-01300-3

**Published:** 2020-08-09

**Authors:** Hye-Young Yun, Jaana Juvonen

**Affiliations:** 1 Department of Psychology, University of Turku, Turku, Finland; 2 Department of Psychology, University of California, Los Angeles, CA USA

**Keywords:** Healthy context paradox, Victimization, Defending, Adolescents

## Abstract

The healthy context paradox—an unexpected pattern in which victims’ psychological adjustment worsens as the overall level of victimization in a classroom or school declines—implies that reducing the frequency of bullying or victimization incidents does not do enough to help victims of bullying. In light of this finding, it is imperative to identify protective factors that alleviate victimization-related distress in the peer ecology. The current study examines classroom-level peer victimization and peer-defending behaviors as moderators of the association between individual-level victimization and psychological adjustment. These classroom-level moderators were tested with a sample of 1373 adolescents (40% girls, *M*_age_: 14 years) from 54 classrooms in South Korean middle schools. Consistent with past findings documenting the healthy context paradox, the results of multilevel modeling indicated that victimized youth experienced a lower level of depressive symptoms in classrooms where victimization was more common. Most importantly, bullied students reported fewer depressive symptoms, on average, in classrooms with relatively high levels of bully-oriented (i.e., confronting the bully), rather than victim-oriented (i.e., comforting the victim), defending behavior. These findings provide a more nuanced understanding of the role of peers’ defending behaviors toward bullied adolescents and have significant implications for anti-bullying interventions.

## Introduction

Experiencing peer victimization (e.g., being pushed, called names, gossiped about) during childhood or adolescence has long-lasting negative effects on social psychological adjustment and is a predictor of persistent victimization (e.g., Brendgen and Poulin 2018; Haltigan and Vaillancourt 2018). Over the last two decades, a number of anti-bullying intervention programs have been developed to decrease peer victimization (e.g., Gaffney et al. 2019; Ttofi and Farrington 2011). Although classrooms and schools with lower rates of bullying and victimization are safer environments for most students (e.g., Cornell et al. 2013; Mehta et al. 2013), paradoxically, recent findings suggest that experiencing bullying in a context with a lower rate of victimization exacerbates the emotional plight of bullied students. This inverse association between victimization-related distress and overall classroom victimization level has been described as the “*healthy context paradox*” (Salmivalli 2018). However, classroom level of peer victimization is unlikely the only contextual factor that affects victimization-related distress (e.g., Chang 2004; Yun 2019). It is therefore critical to identify other contextual factors (besides high levels of victimization) that can help alleviate the distress experienced by bullied youth. This study builds on prior research conducted primarily in European countries to assess whether the healthy context paradox can be replicated in other cultural contexts, in this case among South Korean middle school students. Most importantly, the main goal of the study is to investigate whether bystanders’ involvement in defending behaviors at the classroom level alleviates victimization-related distress.

### CLassroom-level Peer Victimization as a Risk Factor

Anti-bullying intervention research conducted with Dutch and Finnish youth has demonstrated the paradoxical finding of an association between lower classroom rates of peer victimization and greater psychological distress for those who are victimized. For example, those who were or continued to be victimized in schools with anti-bullying interventions reported more depressive symptoms and lower self-esteem than their counterparts in control schools. That is, students who experienced bullying in classrooms or schools with a low level of peer victimization and high centralization of victimization (i.e., only a few individuals are perceived as victims) felt more depressed, on average, than their counterparts in classrooms with higher levels of victimization and lower levels of centralization (Garandeau et al. 2018; Huitsing et al. 2012, 2019). Similarly, non-intervention studies comparing victimized students have found that those in classrooms with lower levels of victimization are more likely to report somatic problems (Gini et al. 2020).

Although researchers have not yet empirically documented the mechanism underlying the healthy context paradox, there are at least two theoretical perspectives that help us understand these paradoxical findings: social comparison theory (Festinger 1954) and attribution theory (Weiner 1986). Social comparison theory postulates that people have a fundamental drive to evaluate themselves by comparing their own experiences to those of relevant others (Festinger 1954). In relation to bullying, this means that when youth see that they are bullied but none (or only a few) of their classmates experience bullying, they feel greater distress (e.g., Bellmore et al. 2004; Nishina and Juvonen 2005). Attribution theory also focuses on how individuals interpret the events they experience. When bullied, victims are likely to ask, “Why me?” Answers to this question can be more or less adaptive. For example, attributing victimization to an internal, stable, and uncontrollable cause (“It’s my fault and I cannot change the way people treat me”: characterological self-blame) is more maladaptive than attributing the experience to an external factors (e.g., “Other kids in this school are mean.”) or internal factors that are unstable and controllable (“I was at the wrong place at the wrong time”: behavioral self-blame) (e.g., Weiner 1986). Moreover, characterological self-blame (internal, stable, and uncontrollable) and behavioral self-blame (internal, unstable, and controllable) vary depending on the rate of victimization. Schacter and Juvonen (2015) found that victimized youth were more likely to endorse characterological self-blaming attributions in schools where victimization was less common, whereas victims were more likely to endorse behavioral self-blame in schools with relatively high levels of victimization. Accordingly, both social comparison and attribution theories imply that the healthy context paradox stems from how victims perceive their own plight relative to the experiences of others.

### CLassroom-level Peer Defending Behavior as a Potential Protective Factor

Contextual factors beyond the prevalence of victimization may also affect victims’ distress. One critical factor is related to bystander responses. Because bullies want to demonstrate their power in front of their peers, they rarely intimidate their targets in the absence of witnesses (see Salmivalli 2014). Indeed, most bullying incidents occur when bystanders are present (Atlas and Pepler 1998; Lynn Hawkins et al. 2001). Given that bullying is a dysfunctional group process involving bystanders as well as the bully and the victim (e.g., Olweus 1993; Salmivalli et al. 1996), the reactions of classmates is an important contextual factor to consider. For example, verbal or nonverbal cues of bystanders (e.g., laughing, assisting, ignoring) can encourage a bully’s behavior. Accordingly, some anti-bullying programs are designed to encourage bystanders to defend victims rather than spur on the bully. Indeed, anti-bullying programs are more effective at decreasing bullying behaviors when bystanders defend victims (e.g., Kärnä et al. 2011; Saarento et al. 2015). Moreover, victims who feel that their peers defended them are better adjusted (i.e., have higher self-esteem) than those who feel undefended (Sainio et al. 2011).

Importantly, defending behaviors in bullying situations take multiple forms. For example, peers can intervene in bullying situations by either confronting the bully or comforting the victim (e.g., Reijntjes et al. 2016; Yun 2019). However, little is known about how these different types of defending behaviors are related to victim distress. By confronting the bullies, peers publicly convey disapproval of their behavior. By contrast, peers can comfort the victim rather discreetly and privately (Yun 2019). Although comforting the victim may be considered a way to alleviate victimization-related distress, it may also convey sympathy, thereby signaling to victims that they cannot do anything about their plight (e.g., Graham 1984; Graham and Barker 1990). As a result, the well-intended behaviors of bystanders may reinforce negative self-perceptions (e.g., “I cannot do anything about being bullied and this will not change”) among the victimized. Thus, whether bystander reactions help the victimized feel less distressed may depend on the specific type of defending behavior.

### The South Korean Middle School Context

While the rates of bullying and victimization in South Korean schools declined in recent years, bullying remains a serious social issue. A recent survey conducted by the Korean Ministry of Education (2019), found that 3.6% of elementary students, 0.8% of middle school students, and 0.4% of high school students had experienced victimization at school (Korean Ministry of Education 2019). As in other countries, Korean youth who are involved in bullying and victimization have a heightened risk of psychosocial maladjustment (for a review, see Hong et al. 2014).

South Korea provides an interesting cultural context because Korean forms of bullying (Wang-ta) have strong conformity characteristics. For example, many students enlist peer norms as justification for excluding and/or ignoring one or two targeted peers (see Yun and Graham 2018; Yun 2019). Moreover, because instruction in South Korean middle schools is classroom based, the same classmates take nearly all of their classes together during a given academic year. In this organizational structure, peer relationships are formed primarily at the classroom level rather than the school level. Therefore, Korean middle school classrooms are well suited to testing contextual effects via the inclusion of cross-level interactions in a multilevel model (see Yun and Graham 2018; Yun 2019).

## Current Study

To gain insight into which contextual factors can help alleviate the distress experienced by victims of bullying, the current study extends the study of the healthy context paradox in two ways. Using a sample of middle school students from South Korea, the first goal is to examine whether the healthy context paradox applies beyond the European cultural context. The second and main goal is to investigate whether different types of defending behaviors (bully-oriented defending and victim-oriented defending behaviors) ameliorate victimization-related depression, regardless of the level of victimization in the classroom.

Using a sample of 1373 South Korean middle school students across 54 classrooms, three kinds of classroom-level variables are calculated: the average level of peer victimization, bully-oriented defending behavior (assertive-defending behavior, e.g., blaming the bully or seeking revenge), and victim-oriented defending behavior (comforting-defending behavior, e.g., comforting the victim) (e.g., Reijntjes et al. 2016; Yun 2019). The prior research suggests two hypotheses: (1) consistent with the healthy context paradox, victimized South Korean youth experience more depressive symptoms in classrooms where victimization is less common, and (2), relative to victim-oriented defending behavior (e.g., comforting the victim afterward), bully-oriented defending behavior (e.g., attacking the bully to defend the victim) has a stronger negative association with distress among bullied students. In other words, bully-oriented defending helps alleviate depressive symptoms of the victimized middle school students.

## Method

### Participants and Procedure

The sample included 1373 students (40% girls, *M*_age_: 14 years) from 54 classrooms (range: 20–32 students; average: 25 students) in six middle schools in Seoul, South Korea. All three grade levels (first: 34%, second: 32%, third: 34%) of middle school (equivalent to sixth, seventh, and eighth grades in the U.S. public school system) were included. The data were collected via paper surveys administered in the spring semester of 2017. To ensure the accuracy of the translation, professional translators in Korea translated and back translated the surveys (from English to Korean and then from Korean back to English), working with bullying experts. The first author attended all classrooms with two research assistants to supervise and to explain the survey to students in a consistent way. Students completed confidential paper surveys in their classrooms during regular school hours; they spent approximately 45 min to an hour completing the survey. Only students who had written parental and youth consent (~98%) were allowed to participate. Before the students began taking the survey, the first author provided them with a clear definition of bullying. In the focal definition, was used the universal agreement, bullying includes three factors: intention, repetition, and power imbalance (e.g., Olweus 1993). Students were asked to report the bullying incidents they had experienced during the current school year.

### Measures

#### Individual-level victimization

Students reported how often they had been the targets of different types of peer victimization (e.g., “made fun of you in front of others”, “hit, kicked, or pushed you”) since the beginning of the school year on a 5-point scale (0 = not at all, 4 = several times a week). Responses to the items were averaged; higher scores indicate greater perceived victimization by peers (*α* = 0.80). This measure, which was created for the UCLA Middle School Diversity Project, relates to other indicators of social and emotional adjustment (see Lanza et al. 2013).

#### Self-reported depressive symptoms

The 7-item short form of the Center for Epidemiologic Studies Depression Scale (CES-D; Radloff 1977) was used to assess depressive symptoms (e.g., “I felt depressed”). Participants were asked to report how often they had experienced each symptom in the past week on a 4-point scale (1 = rarely or none of the time to 4 = almost all the time). The final score was the average of the seven responses, with higher scores indicating more depressive symptoms (*α* = 0.85).

#### Classroom-level peer victimization

To capture the level of victimization in classrooms, individual scores for individual-level victimization were aggregated within each classroom, with higher values indicating higher levels of victimization (range: 1.12–1.87; average: 1.41).

#### Classroom-level Peer Defending Behaviors

To assess the two types of defending behaviors, we used a portion of the Defender and Outsider Roles measure (Yun 2019). Students reported their likelihood of engaging in specific defending behaviors. Three items covered bully-oriented defending behavior (*α* = 0.75; e.g., “I attack the bully to defend the victim”) and three items covered victim-oriented defending behavior (*α* = 0.85; e.g., “I comfort the victim afterward”). Items were rated on a 3-point scale (1 = never, 2 = sometimes, 3 = often). For both bully-oriented and victim-oriented defending behaviors, scores were averaged across the respective items for individuals, and then classroom-level measures were created by averaging all individual-level composite scores for each classroom (bully-oriented, range: 0.22–1.02, M = 0.48, SD = 0.15; victim-oriented, range: 0.40–1.18, M = 0.76, SD = 0.17).

#### Control variables

In addition to gender (at the individual level) and grade (at the classroom level; first grader: 34%, second grader: 32%, third grader: 34%), the neighborhood socio-economic status of the school was used as a control variable at the classroom level. Because Seoul is primarily divided into two regions—Gangbuk (“north of the river”) and Gangnam (“south of the river”)—that have different average income levels, the sample included the same number of schools from each region; three of the six schools (27 classrooms; 53% of participants) were selected from the schools in the northern part of Seoul and three of the six (27 classrooms; 47% of participants) were selected from the schools in the southern part of Seoul.

### Analytic Plan

Hierarchical linear modeling (HLM, Raudenbush and Bryk 2002) was used to test the hypotheses concerning individual- and classroom-level effects. Although classrooms are nested within schools, the current study did not model a three-level HLM because the number of schools was not sufficient to warrant analysis at the school level (Maas and Hox 2005). HLM simultaneously assesses relations within and between hierarchical levels and accounts for the shared variance in hierarchically structured data. This approach prevents the incorrect partitioning of variance into variables and accounts for the increased risk of making a Type 1 error (Raudenbush and Bryk 2002). Because the participants who dropped out provided partial data (0.1–0.4%), they were included in the analysis based on the assumption of missing completely at random (MCAR) as supported by the expectation-maximization test. Missing data, including blanks or missing data codes in the raw data, were handled in the HLM software package via listwise deletion at the first level by maximum likelihood estimation routines. Based on recommendations developed by Raudenbush and Bryk (2002), the relative proportion of variance explained by the addition of each level of predictors was compared by fitting three steps—(1) an unconditional model with no predictors, (2) a conditional model with only individual-level predictors, and (3) a conditional model with both individual- and classroom-level predictors. Step 1 calculates the intraclass correlation; the difference between the coefficients in Steps 1 and 2 is the relative proportion of variance explained by all student-level predictors; and the difference between the coefficients in Steps 2 and 3 is the relative proportion of variance explained by the classroom-level predictors. The main contextual moderator hypotheses were tested by including cross-level interaction terms. That is, interaction terms between individual-level victimization (Level 1) and classroom-level (Level 2) indicators (peer victimization, bully-oriented defending, and victim-oriented-defending) were tested.

## Results

The means, standard deviations (SD), and correlation coefficients for the student-level variables of main effects are presented in Table 1. As expected, individual-level victimization was positively related to depressive symptoms. Individual-level victimization was also related to classroom-level victimization. The two defending behaviors were correlated, suggesting that students who engaged in one type of defending behavior also engaged in the other type.

**Table 1:** Correlations, means, and standard deviations of study variables for individual- and classroom-level models

Variables	1	2	3	4	5
1. Individual-level victimization	1				
2. Depressive symptoms	0.30**	1			
3. Classroom-level peer victimization	0.34**	0.07**	1		
4. Classroom-level bully-oriented defending behavior	0.14**	0.01	0.41**	1	
5. Classroom-level victim-oriented defending behavior	0.10*	0.03	0.29**	0.66**	1
Mean	1.42	1.76	1.41	0.48	0.76
Standard deviation	0.48	0.64	0.16	0.15	0.17

*Note*. Individual-level victimization and depressive symptoms are individual-level variables; peer victimization, peer bully-oriented defending behavior, and peer victim-oriented defending behavior are classroom-level variables

*p < 0.05; **p < 0.01; ***p < 0.001

### Step 1: The Unconditional Model

An unconditional model was conducted to assess the intraclass correlation (ICC) or the proportion of variance in depressive symptoms due to classroom-specific factors. Without any predictors on either Level 1 or Level 2, the estimated variance within classrooms was, *σ*^2^ = 0.42564, and the variance between classrooms was, τ_00_ = 0.00786. While the ICC value was not high (0.03), the chi-square test of between-classroom variance showed meaningful variability in depressive symptoms across the 54 classrooms, *χ*^2^(53) = 58.81, *p* = 0.03.

### Step 2: The Main Effects Model

To explore depressive symptoms as a function of individual victimization experiences, the individual-level victimization was added to the model while controlling for gender. Following Bryk and Raudenbush’s recommendations (2002), individual-level victimization was centered around the group mean to produce more accurate estimates of the intercepts, while gender was coded as a dummy variable (gender: boy = 0; girl = 1). The results of the estimation of the main effects model showed that individual-level victimization (*b* = 0.35, *SE* = 0.04, *p* < 0.001) had a positive effect on depression symptoms. Further, girls reported higher levels of depressive symptoms than boys (*b* = 0.49, *SE* = 0.05, *p* < 0.001) (see Model 2 in Table 2). For the random effects model, the statistical significance of the variability in the slopes for the individual-level predictors on depressive symptoms was explored. Because none of the predictors had significant variability in slopes, the terms for random slopes were removed from each of these predictors in Model 3. The individual-level residual variance, $\widehat \sigma ^2$, in Model 2 decreased relative to the residual variance in the unconditional Model 1. The proportional decrease in variance (0.17) means that 17% of the variability in reported depressive symptoms can be attributed to the full set of individual-level predictors entered in this model. However, between-classroom variance in the relation between individual-level victimization and depressive symptoms was significantly greater in the model with gender control than in the unconditional model (*χ*^2^(53) = 64.30, *p* < 0.01), which implies that there is also substantial variability across classrooms in the effects of individual-level victimization on depressive symptoms.

**Table 2:** Fixed effects and random effects of the hierarchical linear model predicting depressive symptoms

	Model 1	Model 2	Model 3	Model 4
	Coeff. (SE)	Coeff. (SE)	Coeff. (SE)	Coeff. (SE)
Fixed effects				
Intercept	1.78 (0.03)***	1.64 (0.03)***	1.67 (0.05)***	1.67 (0.06)***
Individual level (IL)				
Victimization		0.35 (0.04)***	0.49 (0.05)***	0.50 (0.04)***
Gender		0.49 (0.05)***	0.36 (0.04)***	0.34 (0.04)***
Classroom level (CL)				
School region			−0.02 (0.05)	−0.02 (0.06)
First grade			−0.01 (0.06)	−0.01 (0.06)
Second grade			−0.03 (0.06)	−0.05 (0.06)
Peer victimization			0.51 (0.21)	0.50 (0.04)***
Bully-oriented defending behavior				−0.15 (0.16)
Victim-oriented defending behavior				0.64 (0.37)
Interaction effects				
IL Victimization × CL Peer victimization			−2.19 (0.51)*	
IL Victimization × CL Bully-oriented defending behavior				−1.20 (0.62)*
IL Victimization × CL Victim-oriented defending behavior				0.12 (0.28)

*Note*. School Region: southern region of Seoul = 0; northern region of Seoul = 1. Gender: boy = 0; girl = 1. Grade: first grade and second grade are dummy variables (third grade is the reference group). Individual-level victimization was group-mean centered, and classroom-level peer victimization, peer bully-oriented defending behavior, and peer victim-oriented defending behavior were grand-mean centered

*Coeff.* coefficient, *SE* standard error

*p < 0.05; **p < 0.01; ***p < 0.001

### Step 3: The Cross-level Interaction Model

To test the moderator hypothesis about the effects of classroom-level contextual factors, cross-level interactions between classroom-level variables and individual-level victimization were added. As shown in the results of Model 3 in Table 2, there was a significant interaction between individual-level victimization and classroom-level peer victimization (*b* = −2.19 SE = 0.51, *p* < 0.05). To further probe this interaction, follow-up tests of simple slopes were conducted for individuals in classrooms that were either high (+1 SD) or low (−1 SD) on overall peer victimization. Consistent with the hypothesized healthy context paradox, the association between individual-level victimization and depressive symptoms was significantly stronger for individuals in classrooms with lower levels of peer victimization than for students in classrooms with higher levels of peer victimization.

**Fig. 1:**
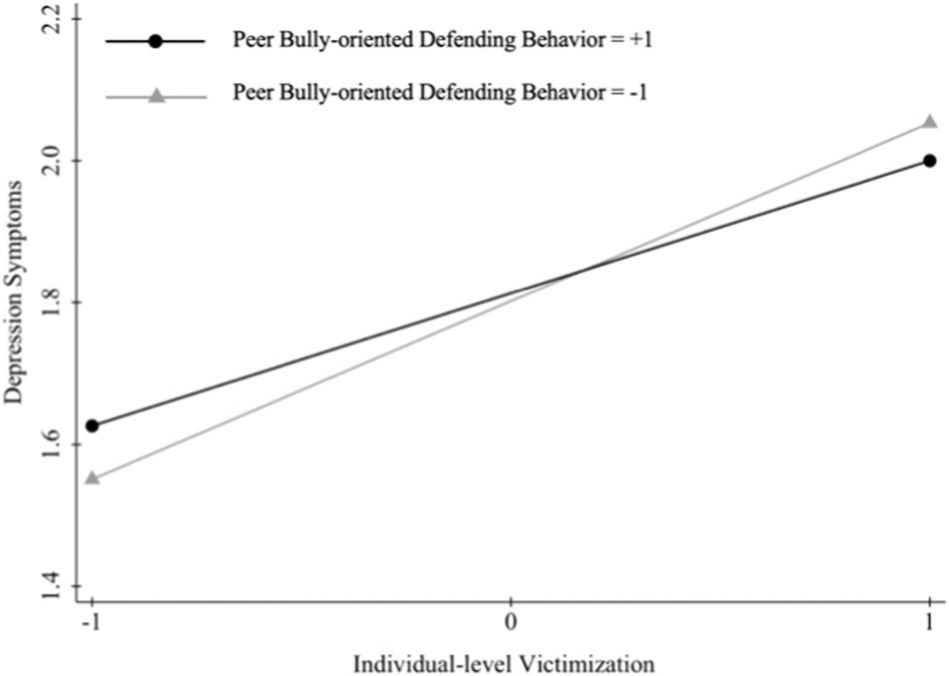
Associations between individual-level victimization and depressive symptoms as a function of classroom-level peer bully-oriented defending behavior. Note. X-axis ranges from −1 SD to +1 SD of individual-level victimization with simple slopes plotted at low (−1 SD) and high (+1 SD) levels of peer bully-oriented defending behavior

Turning to the moderating effects of peer defending behaviors, classroom-level peer bully-oriented and victim-oriented defending behaviors were added to the model, controlling for classroom-level peer victimization. As seen in the results presented in Model 4 in Table 2, while students reported more depressive symptoms when they experienced increased victimization, this link was weaker for students in classrooms with higher (+1 SD) average levels of bully-oriented defending behavior relative to those in classrooms with lower (−1 SD) average levels (*b* = −1.20, *SE* = 0.62, *p* < 0.05) (see Fig. 1). In contrast, the interaction between individual-level victimization and victim-oriented defending behavior was not statistically significant.

The proportion of variation explained by the addition of classroom-level predictors can be calculated by comparing the results of Models 2 and 4, which include the same set of individual-level predictors. This calculation shows that the proportion of variance explained by classroom-level factors is 0.40, which means that adding classroom predictors to the model reduced classroom variability in outcomes by 40%.

### Sensitivity Analyses

Due to the organization of instruction in South Korean middle schools, the current study focused on classroom-level, rather than school-level, contextual factors. Although classrooms are nested within schools, we chose not to use a three-level model because there were not enough schools to warrant analysis at the school level (Maas & Hox 2005). As mentioned above, Seoul is primarily divided into two regions, Gangbuk (“north of the river”) and Gangnam (“south of the river”), and we selected the same number of schools from each region. Thus, school region (i.e., Gangbuk, Gangnam) was controlled in the main analysis. Despite rigorous statistical analysis using a large number of classrooms with participants evenly nested in classrooms (average: 25 students; range: 20–32 students), it may be difficult to completely ignore school-level variance. Thus, to ensure that the findings are not sensitive to school-level variance, a sensitivity analysis was conducted via an alternative three-level model for exploratory purposes.

Following the main analysis steps described above, an unconditional model that did not include predictor variables was examined first to estimate the proportion of variance across each of the three levels. The proportion of variance for students within classrooms, between classrooms within schools, and between schools was 0.443, 0.019, and 0.005, respectively. Moreover, there was no statistically significant variance between schools (*χ*^2^(5) = 11.190, *p* > 0.500). Next, a fully conditional three-level model was run that included the same predictors at the student- and classroom-levels as in the original model but also included aggregated school-level peer victimization and peer-defending behaviors at Level 3. All results were quantitatively similar to those reported above at Level 1 and Level 2, and there were no statistically significant school-level effects on the association between victimization and depressive symptoms, which indicates that the rest of the predictors (at the individual and classroom levels) remained significant when school-level factors were controlled. This alternative model potentially implies that the above findings regarding the effect of peer-related contextual factors on victimization-related distress are not sensitive to the school-level variation in the South Korean middle school context. Thus, victimization-related psychological adjustment may be more closely related to the behavior of classmates, who can directly influence bullying incidents in the classrooms, than to peer behavior at the school level.

## Discussion

Bullying researchers and educational practitioners have made important progress in reducing the level of bullying and victimization in schools and classrooms (e.g., Huitsing et al. 2020; Kärnä et al. 2011, 2013). Although it is good news that anti-bullying intervention programs are effective, it is disconcerting that reductions in rates of bullying and victimization can elevate the distress of those who are bullied. Given that it is not possible to eradicate bullying completely, it is vital to identify protective contextual factors that help the bullied. The current study sheds light on these protective classroom-level factors by suggesting that over and beyond the classroom level of victimization implicated in the healthy context paradox, having peers who stand up to bullying is critical for victims to feel less depressed. The current findings are novel—they provide a more nuanced understanding of the role of defending behaviors and thus have significant implications for anti-bullying interventions.

Most importantly, the current study expands the research on classroom-related contextual moderators by focusing on bystander behaviors, examining whether two types of defending behaviors alleviate victims’ distress. The most novel finding of the current study is the classroom-level moderating effect of peer bully-oriented defending behavior on the association between victimization and distress. As expected based on attribution theory (e.g., Graham and Juvonen 1998; Weiner 1986), the association between victimization and depression was attenuated in classrooms where more classmates confronted bullies directly in bullying situations. This finding held across classroom levels of peer victimization. This pattern might occur because, when classmates’ defending behavior focuses on bullies, victims are less likely to blame themselves and therefore experience fewer depressive symptoms. Moreover, prior research indicates that students who engage in bully-oriented defending behavior are perceived as more popular and report a higher sense of self-efficacy related to stopping bullying (Yun 2019). Being defended by peers who are more influential in the peer group may make victims feel more protected and more likely to believe that the bullying situation can change.

In contrast to bully-oriented defending behavior, victim-oriented defending behavior by peers was not related to victims’ depression. Because comforting victims does not address the bullying behavior itself, victims may not perceive comforting behavior as helpful to their plight, and may even consider it unsolicited support (for a review see Barrera 2000). Moreover, from an attributional perspective (e.g., Graham 1984; Graham and Barker 1990), the well-intended comforting of victims, especially if it is unsolicited, may reinforce the idea that they cannot do anything about their plight themselves. Accordingly, being comforted by peers, while desirable (Yun 2019) in the sense that such behaviors convey kindness and care, may do little to change victims’ causal interpretations, which are in turn related to feelings of depression. In sum, the study showed that not all types of peer defending behaviors have positive consequences for victims’ interpretations of the bullying situation.

The second contribution of the study is the replication of the healthy context paradox among non-European adolescents, specifically in the South Korean middle school context. Consistent with previous results for Dutch, Finnish, and Italian youth (i.e., Garandeau et al. 2018; Gini et al. 2020; Huitsing et al. 2012, 2019; Kaufman et al. 2018), victimization-related distress was heightened in classrooms with a lower level of peer victimization. Because victims in a classroom with a lower level of victimization are less likely to have peers with similar victimization experiences (e.g., Schacter and Juvonen 2018), they might be more likely to believe that the causes of their mistreatment are internal, stable, and uncontrollable (i.e., characterological self-blame; Graham and Juvonen 1998; Weiner 1986). This belief, in turn, is related to increased feelings of depression (e.g., Schacter and Juvonen 2015).

### Limitations and Future Directions

The findings of the current study should be interpreted carefully in light of certain limitations. First, the victimization measure has certain constraints. The victimization measure was based on self-reports. While self-reports present a unique individual-level view of situations that peers either cannot observe or may not consider bullying behavior (e.g., Pellegrini 2001), the resulting data are based on the potentially biased view of only one participant. In addition, the study did not differentiate between bully-victims (who bully others and are also victimized themselves) and non-aggressive victims. Future studies should disaggregate these two groups.

Second, the two types of defending behavior were also measured via self-reports. Because comforting-defending behavior is a relatively personal behavior, Yun (2019) assumed that a self-rating would capture the two types of defending behaviors more effectively than a peer-rating. However, given that socially desirable responding is most likely to occur in response to socially sensitive questions, such as those asking about prosocial behaviors (King and Brunner 2000), students may overestimate their own defending behaviors in bullying situations. To reduce subjectivity, social desirability bias, and related errors, future studies that incorporate peer and teacher ratings, in addition to self-ratings, are warranted.

Third, because the data set used for current study did not contain information on whether victims perceived that they were defended by peers (a bullied student might not perceive being defended even if the classroom has a high level of peer-defending behaviors) or how they interpreted different types of defending behaviors, we could not provide direct evidence of the proposed attributional mechanism. To test the interpretations associated with victimization-related distress (e.g., characterological self-blame), future studies must gain insight into the relevant cognitive processes.

Fourth, this analysis of cross-sectional data did not allow a test of whether students experienced depressive symptoms because they were bullied by peers or whether students who were already depressed were more likely to experience victimization. Longitudinal studies are needed to examine the causal direction of the relationships between victimization, classroom-level moderators, and depressive symptoms.

Lastly, because this study was conducted in a racially homogeneous South Korean middle school context, the results cannot be generalized to a context with more racially and ethnically diverse schools and classrooms (e.g., the United States). Given that students who are members of numerical minority ethnic groups are particularly vulnerable to being bullies, whereas classroom and school diversity are protective factors (Juvonen et al. 2013), future research should examine how individual-level race/ethnicity and contextual racial/ethnic diversity moderate the associations between victimization, psychological adjustment, and contextual victimization as well as peers’ defending behaviors. Moreover, because South Korea has been identified as a strongly collectivistic culture (Hofstede 2016), this study assumed that Korean classrooms have strong conformity characteristics, however, we did not assess collectivism. Thus, future studies should investigate that whether our novel finding that bully-oriented defending behavior is a protective factor is culturally specific, and should consider other cultural factors as well.

### Implications and Challenges for Anti-bullying Interventions

The current findings have significant implications for understanding how classroom contextual factors can both harm and bolster the psychological adjustment of bullied middle school students. The results underscore the need to redefine a healthy school environment: While it is clearly true that schools should keep pursuing the reduction of victimization and bullying in classroom and school contexts, making these contexts healthy for *all* students will require identifying additional ways to effectively protect the remaining bullied youth. Moreover, the current study also highlights the importance of determining which types of defending behavior are more helpful in alleviating distress. The findings do not imply that victim-oriented defending behavior is not important; rather, they suggest that it is especially important to empower classmates to confront bullies. However, accomplishing this is a challenging goal for an intervention. Because bullies are frequently considered “cool” and powerful, particularly during adolescence, specifically during middle school (e.g., Juvonen et al. 2003; LaFontana and Cillessen 2002; Vaillancourt and Hymel 2006; Yun and Graham 2019), defending behavior can increase a defender’s risk of being a future target of bullying (Yun and Graham 2018). As a result, adolescents, who have a strong need to affiliate with and belong to their peer group (Brown and Larson 2009), may hesitate to stand up to bullies. Thus, advancing anti-bullying interventions will require the development of a feasible strategy for mobilizing students to shift the power dynamic by joining together, rather than engaging alone, to safely challenge bullies.

## Conclusion

While it is important to continue efforts to reduce bullying and victimization, these efforts alone will not improve the mental health of the remaining victims. Hence, it is imperative that researchers identify factors that make classrooms and schools safe for all students. The current study focused on one set of classmate behaviors known to be critical: coming to the aid of bullied students. The findings underscore the nuanced differences between types of defending behaviors by suggesting that publicly objecting to and challenging bullying behaviors is more effective at reducing victimization-related depression than offering comfort to victims. Although these findings must be replicated before they can be used to inform interventions, they raise a number of questions. Most importantly, why is public objection to bullying a powerful alleviator of the distress of the bullied, while well-meaning comforting of victims is not? These issues must be understood first and foremost from the perspective of the victims themselves—just as social comparisons can highlight victims’ relative standing compared to their peers, attributional inferences about classmates’ reactions can provide critical information about the ways victims interpret helping behaviors. Although this study was conducted in South Korea, which is still a racially homogeneous country, identifying both universal and nation-specific protective factors will greatly improve future anti-bullying interventions and help policymakers and practitioners across the globe address school bullying.
